# Performance of a dipstick dye immunoassay for rapid screening of *Schistosoma japonicum *infection in areas of low endemicity

**DOI:** 10.1186/1756-3305-4-87

**Published:** 2011-05-20

**Authors:** Jing Xu, Ting Feng, Dan-Dan Lin, Qi-Zhi Wang, Li Tang, Xiao-Hua Wu, Jia-Gang Guo, Rosanna W Peeling, Xiao-Nong Zhou

**Affiliations:** 1 National Institute of Parasitic Diseases, Chinese Center for Disease Control and Prevention, WHO Collaborating Center for Malaria, Schistosomiasis and Filariasis, Shanghai 200025, People's Republic of China; 2 Provincial Institute of Parasitic Diseases, Nanchang, Jiangxi 330046, People's Republic of China; 3 Provincial Institute of Parasitic Diseases, Hefei, Anhui 230061, People's Republic of China; 4 Provincial Institute of Parasitic Diseases, Wuhan, Hubei 430070, People's Republic of China; 5 London School of Hygiene and Tropical Medicine, London, UK

## Abstract

**Background:**

The dipstick dye immunoassay (DDIA), recently commercially available in the People's Republic of China (P.R. China), is a rapid and simple test to detect human antibodies against *Schistosoma Japonicum*. Its performance and utility for screening schistosome infection in low endemic areas is little known. We therefore carried out a cross-sectional survey in seven villages with low endemicity of schistosomiasis in P.R. China and assessed the performance and utility of DDIA for diagnosis of schistosomiasis. Stool samples were collected and examined by the Kato-Katz method and the miracidium hatching technique. Serum samples, separated from whole blood of participants, were tested by DDIA.

**Results:**

6285 individuals aged 6-65 years old participated in this study, with a prevalence of schistosomiasis of 4.20%. Using stool examination as a gold reference standard, DDIA performed with a high overall sensitivity of 91.29% (95% CI: 87.89-94.69%) and also a high negative predictive value, with a mean value of 99.29% (95% CI: 98.99-99.58%). The specificity of DDIA was only moderate (53.08%, 95% CI: 51.82-54.34%). Multivariate analysis indicated that age, occupation and history of schistosome infection were significantly associated with the false positive results of DDIA.

**Conclusions:**

DDIA is a sensitive, rapid, simple and portable diagnostic assay and can be used as a primary approach for screening schistosome infection in areas of low endemicity. However, more sensitive and specific confirmatory assays need to be developed and combined with DDIA for targeting chemotherapy accurately.

## Background

Schistosomiasis japonica is one serious infectious disease, draining the economic and social development in the People's Republic of China (P.R. China) [1]. An estimated 100 million people were at risk of contracting schistosomiasis and 11.6 million were infected in 12 endemic provinces in P.R. China in the mid 20^th ^century [1,2]. With continuous national programs being implemented in P.R. China, great achievements have been made in the control of schistosomiasis. The prevalence and intensity of *Schistosoma japonicum *(*S. japonicum*) infection have decreased dramatically. Most counties have reached the criteria of infection control (human prevalence less than 5% ), while in many others, transmission control (human prevalence less than 1%) or even transmission interruption (no case found in five consecutive years) has been achieved [3]. These different endemic levels increase the demand of sensitive and cost-effective diagnosis for accurate identification of schistosomiasis cases, followed by treatment of individuals and/or communities, and evaluation of intervention efficacy as the control goal is still to reduce the prevalence to a sustainable low level [4].

Due to lack of other pragmatic diagnostic methods, the Kato-Katz method is still the most widely used for direct diagnosis of intestinal schistosomiasis in P.R. China, although it fails due to its insensitivity in regions of low endemicity and light infections, especially when only one stool specimen is used for diagnosis [5,6]. Combination of the Kato-Katz method and the miracidium hatching technique could decrease the misdiagnosis of patients, but the performance of the latter is prone to be affected by various factors such as temperature and quality of water [7-10]. Furthermore, direct stool examinations on a population level to find a few cases will be costly and are not appropriate in areas of low endemicity. And the compliance of residents to provide stool specimens were also decreased year after year [11,12]. To overcome these shortcomings, a two-step method has been implemented for guiding chemotherapy, estimation of endemic status, and assessment of intervention efficiency in the schistosomiasis control programs in P.R. China, with antibody-based immunoassay as a primary approach for screening the human population due to its higher sensitivity and simple operational characteristics. Only antibody positive cases are followed by stool examination to make sure whether they are currently infected with schistosomes. [2,13-16].

Facilitated and improved by advances in immuno-labeling techniques, there are several kinds of immunoassays for diagnosis of schistosome infection which have been developed and implemented for screening, such as the circumoval precipitin test (COPT), indirect hemagglutination test (IHA), enzyme-linked immunosorbent assay (ELISA), etc. [13,17,18]. But the intrinsic features of these assays, such as complex and time-consuming procedure, needs of extra instruments etc., have limited their use on a large scale in field settings especially in areas of low endemicity with limited resources [19]. There is an increased need for sensitive, rapid, simple and inexpensive assays for screening of schistosomiasis, especially in the case of on-the-spot surveys in low endemicity areas.

With the growing interest in the use of rapid diagnostic test for schistosome infection, dipsticks, based on lateral immunochromatographic flow method, have been used to detect circulating cathodic antigen (CCA) of *Schistosoma mansoni *infection and proved to be an alternative methodology for estimating infection prevalence and intensity [20]. Recently, a rapid and simple test named dipstick dye immunoassay (DDIA) has been made commercially available in P.R. China market to detect human antibodies against schistosomes. This assay can be done in 5-10 minutes per test without additional equipment except a micropipettor [21]. Laboratory-based evaluation and field trials proved that DDIA performed with high sensitivity in areas with high endemicity and high specificity in areas free of schistosome infection [22,23]. In this study, we report on the performance of DDIA compared with stool examination and evaluate its efficacy as a primary approach for screening the population in seven villages of low endemicity.

## Methods

### Study areas, population and sample collection

A cross-sectional survey was carried out in seven separate administrative villages from three provinces along Yangtz River in China: Caohui, Jingtou and Xinhua villages located in Jiangxi Province, Longshang, Tieguai and Yuye villages from Anhui province, and Hebei village administrated by Hubei province. We invited all individuals aged from 6-65 years old in each village to participate in our study which was performed in October and November 2008.

Demographic data of individuals, including age, gender, occupation, birth date and history of schistosome infection and other related diseases, were recorded by a structured questionnaire. Each participant was asked to provide one stool sample over 50 g and one blood sample over 250 μl through finger prick. The samples were taken to local schistosomiasis stations, where stool examinations and DDIA tests were performed by laboratory staff.

### Ethical statement

Written informed consent was obtained from all adult participants and from the parents or legal guardians of children. The study was approved by the ethical committee of National Institute of Parasitic Diseases, China Center for Disease Control and Prevention. Any participant found to be parasitologically positive received anthelmintic treatment except for those with contraindications.

### Stool examination

Each stool specimen was processed using the Kato-Katz thick smear method and the miracidium hatching technique without knowledge of the infection result of previous diagnostics.

Kato-Katz technique was performed as previously described to detect *S. japonicum *eggs in stool samples [24]. Briefly, three slides from a single stool specimen were prepared using a standard template (41.7 mg per smear). All slides were read 12-48 h after their initial preparation by two qualified technicians, who were unaware of the subjects' medical status, results of miracidium hatching technique and DDIA detection. The number of eggs in each slide was counted and recorded. Infection intensity of patients was expressed as eggs per gram of feces (EPG), using arithmetic mean of all eggs counts obtained from three slides multiplied by 24.

Miracidium hatching technique was performed as follows: 30 g of feces were homogenized in dechlorinated water and then filtered over double-layered gauze to remove large detritus, repeating the filtration until a clear supernatant liquid was obtained. The suspension was then transferred to a volumetric flask and hatched in 26-30°C in an illumination incubator. Observations were taken 4, 8 and 12 hours after hatching with the naked eye or by means of a magnifying glass. The result was defined as positive if moving miracidia in the supernatant were observed [25]. The infection intensity of cases that were considered negative by the Kato-Katz method but positive by the miracidium hatching technique was regarded as one EPG.

Combining the results of the Kato-Katz method and the miracidium hatching technique, a patient was defined as positive if positive results were obtained with either parasitological technique and as negative if negative results were obtained with both parasitological methods. The infection intensity of cases was divided into three categories as *S. mansoni *defined by World Health Organization: light infection (1-99 EPG), moderate infection (100-399 EPG) and heavy infection (≥400 EPG) [26].

### DDIA

Serum was separated from the whole blood sample and was tested, without prior knowledge of the results of stool examination. DDIA kits were supplied by Wuxi Saide Medical Technology Co. Ltd, Jiangsu, P.R. China (Product Lot: 0809101). The test uses soluble antigen extracted from *S. japonicum *eggs as the probe to detect antibodies against *S. japonicum *in human blood [21].

Serological tests were performed according to the instructions supplied by the manufacturer. Briefly, a drop of 50 μl blue colloidal dye-labeled soluble egg antigens (SEA) solution from the buffer bottle was added into a polyvinyl chloride (PVC) well and 20 μl of serum specimen was added. The solution in the well was mixed lightly for about one minute. A dipstick was then inserted into the well. The result was read after the solution was absorbed completely. The appearance of two blue bands on the dipstick indicated a positive reaction, and the appearance of a single blue band in the control position indicated a negative reaction [21].

### Statistical analysis

In the field survey, only data from subjects who accepted stool examination and serological tests were used for the analysis. The combination of the Kato-Katz method and the miracidium hatching technique was considered the diagnostic gold standard. And the diagnostic performance of DDIA was assessed by calculating sensitivity, specificity, positive predictive value (PPV) and negative predictive value (NPV). Sensitivity was calculated as the percentage of participants who were seropositive among those who were positive by stool examination. Specificity was expressed as the percentage of individuals who were seronegative among those who were stool examination negative. PPV was calculated to express the screening efficiency of DDIA by determining the percentage of those who were stool examination positive among those who were seropositive, and NPV was expressed as the percentage of individuals who were stool examination negative among those who were seronegative. 95% confidence intervals (CI) were calculated for all diagnostic scores. The comparison between groups was analyzed by the Chi-squared test. The relationships between the prevalence and characteristics of DDIA were analyzed by Pearson's correlation analysis. We judged a *P *value of less than 0.05 to be significant.

For analyzing factors associated with false positive DDIA results, bivariate analysis of various variables was done to identify factors associated with false positivity of DDIA using crude odds ratios (OR) and their 95% CI. The analysis was restricted to individuals for whom complete sociodemographic and clinical data were available. All variables that were significant based on bivariate analysis (*P *< 0.05) were included in a multivariate model. Adjusted OR and their 95% CI were calculated. Factors that remained independently associated with the outcome at a *P *value of <0.05 were retained. All analyses were performed using the SPSS (Statistical Products & Service Solutions) package for windows (SPSS Inc., Chicago, USA, version 13·0).

## Results

### Demographic data

A total of 7,996 people in seven villages voluntarily participated in field survey. 78.60% (6285/7996) of them had completed demographic information and also provided both a single fecal specimen for stool examination and a blood sample for serum for DDIA test. The whole valid population was composed of 48.23% (3031/6285) men and 51.77% (3254/6285) women, with a mean age of 38.32 years old (Standard Error (S.E.): 17.71). Most participants were farmers (62.86%, 3951/6285) and students (21.70%, 1364/6285).

### Endemic areas were characterized with low endemicity of schistosomiasis

The prevalence of *S. japonicum *infection determined by stool examination was in the range of 0.38% - 8.23% in seven communities, while geometric mean EPG (± S.E.) of cases ranged from 5.98 ± 7.90 to 45.04 ± 4.98 (Table 1). For the whole population, the prevalence of schistosomiasis was 4.20% (264/6285). Among the positive individuals, 84.47% (223/264) were classified as having a low intensity infection (1-99 EPG), 11.74% (31/264) had moderate infections (100-399 EPG), and 3.79% (10/264) had heavy infections (≥400 EPG). 4.44% (279/6285) of the whole population were infected with soil-transmitted helminthes, mainly *Ascaris lumbricoides*, *Trichuris trichiura *and hookworm. These data indicated that the study areas were characterized by low prevalence and intensity of schistosome infection.

**Table 1:** Prevalence and intensity of *S.japonicum *infection detected by stool examination

Village	No. of examined	No. of positives	Prevalence (%)	Geometric mean EPG for infected (Mean ± S.E.)	Other helminthes prevalence rate % (No. of positives)
Longshang	1044	4	0.38	5.98 ± 7.90	0.00 (0)
Hebei	887	16	1.80	10.48 ± 7.26	0.11 (1)
TieGuai	859	22	2.56	14.73 ± 3.18	1.40 (12)
Xinhua	922	46	4.99	26.05 ± 6.24	2.93 (27)
Yuye	824	44	5.34	13.75 ± 4.21	1.09 (9)
Caohui	826	56	6.78	22.02 ± 3.56	20.10 (166)
Jingtou	923	76	8.23	45.04 ± 4.98	6.93 (64)

Total	6285	264	4.20	23.35 ± 5.05	4.44 (279)

### Performance of DDIA compared with stool examination

The results of DDIA compared with those of stool examination are shown in Table 2. Using the results of stool examination as the gold standard, sensitivity, specificity, PPV and NPV of DDIA were calculated.

**Table 2:** Performance characteristics of DDIA compared with stool examination

Criterion assessed	Longshang	Hebei	TieGuai	Xinhua	Yuye	Caohui	Jingtou	Total
No. of true positives	3	15	21	41	40	51	70	241
No. of false positives	579	186	517	383	490	359	311	2825
No. of true negatives	461	685	320	493	290	411	536	3196
No. of false negatives	1	1	1	5	4	5	6	23
Sensitivity [%, 95% CI]	75.00 [32.56-100.00]	93.75 [81.89-100.00]	95.45 [86.75-100.00]	89.13 [80.14-98.13]	90.91 [82.41-99.40]	91.07 [83.60-98.54]	92.11 [86.04-98.17]	91.29 [87.89-94.69]
Specificity [%, 95% CI]	44.33 [41.31-47.35]	78.65 [75.92-81.37]	38.23 [34.94-41.52]	56.28 [52.99-59.56]	37.18 [33.79-40.57]	53.38 [49.85-56.90]	63.28 [60.04-66.53]	53.08 [51.82-54.34]
PPV [%, 95% CI]	0.52 [0-1.10]]	7.46 [3.83-11.10]	3.90 [2.27-5.54]	9.67 [6.86-12.48]	7.55 [5.30-9.80]	12.44 [9.24-15.63]	18.37 [14.48-22.26]	7.86 [6.91-8.81]
NPV [%, 95% CI]	99.78 [99.36-100.00]	99.85 [99.57-100.00]	99.69 [99.08-100.00]	99.00 [98.12-99.87]	98.64 [97.32-99.96]	98.80 [97.75-99.85]	98.89 [98.01-99.77]	99.29 [98.99-99.58]

CI: Confidence interval

**Figure 1:**
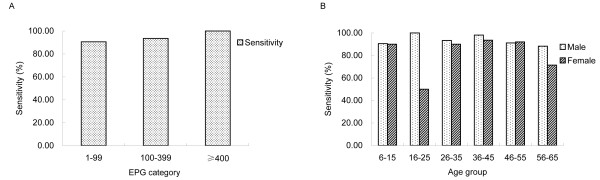
**Sensitivity of DDIA across (A) infection intensity categories (B) age and gender strata**.

The sensitivity of DDIA was high with a mean value of 91.29% (95% CI: 87.89-94.69%) for whole population, with a range of 75.00% (95% CI: 32.56-100.00%) to 95.45% (95% CI: 86.75-100.00%) across the seven villages while no significant difference existed between any two villages (χ^2 ^= 2.28, *P *> 0.05). When infected individuals were divided into infection intensity categories as light, moderate and heavy infection (EPG < 100, 100-399, ≥400, respectively), the sensitivity of DDIA increased with infection intensity, but no significant difference was detected (χ^2 ^= 1.29, *P *> 0.05) (Figure 1A). Among the false negative cases determined by DDIA, 91.30% (21/23) of them had an infection intensity lower than 100 EPG. Sensitivity did not differ significantly between gender and age categories (Figure 1B).

**Figure 2:**
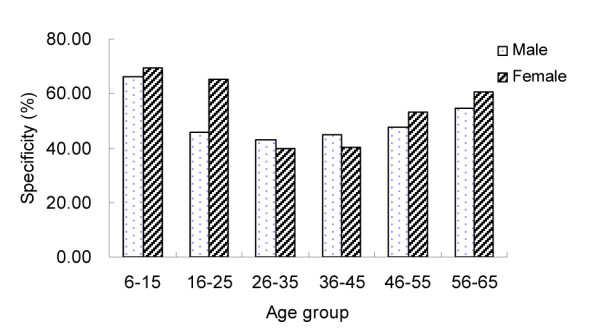
**Specificity of DDIA by age and gender groups**.

The specificity of DDIA varied significantly from 37.18% (95% CI: 33.79-40.57%) to 78.65% (95% CI: 75.92-81.37%) among seven communities (χ^2 ^= 452.87, *P *< 0.05), with an overall specificity of 53.08% (95% CI: 51.82-54.34%) (Table 2). Specificity did not vary by gender (χ^2 ^= 0.83, *P *> 0.05). However, specificities differed markedly among age groups (χ^2 ^= 221.37, *P *< 0.05), being higher in the youngest and eldest groups but lowest in the age groups corresponding to 26-35 and 36-45 years old (Figure 2).

The PPV of DDIA was very low with the mean value of 7.86% (95% CI: 6.91-8.81%), ranged from 0.52% (95% CI: 0-1.10%) to 18.37% (95% CI: 14.48-22.26%) in the seven surveyed communities. Pearson's correlation coefficient (r) between the prevalence of schistosome infection and PPV of DDIA was 0.92 (*P *< 0.05), suggesting that a strong positive correlation exists between the prevalence of schistosome infection and PPV of DDIA at community level. The NPV of DDIA was high in the range of 98.64% (95% CI: 97.32-99.96%) to 99.85% (95% CI: 99.57-100.00%) with an average value of 99.29% (95% CI: 98.99-99.58%). A significant negative correlation was detected between the prevalence of schistosome infection and NPV of DDIA with an r value of -0.88 (*P *< 0.05).

### Factors influencing the false positive DDIA results for schistosome infection

The results of bivariate logistic regression analysis were listed in Table 3. Individuals aged greater than 15 years old were more likely to have false positive results of DDIA with crude OR in the range of 1.54 (95% CI: 1.31-1.82) - 2.99 (95% CI: 2.44-3.67), (*P *< 0.001), compared to those individuals aged 6-15 years old. Individuals whose reported occupation was 'fisherman' or 'boatman' (crude OR 2.23, *P *< 0.001), and students (crude OR 0.52, *P *< 0.001) were significantly associated with a false positive DDIA test, as compared with farmers. The history of schistosome infection was strongly associated with false positive DDIA results (crude OR 2.70, P < 0.001).

**Table 3:** Association of demographic and diseases factors with the false positive results of DDIA

Variables	No. (%) of participants with the following DDIA results		Crude OR (95% CI)	*P *value	Adjusted OR (95% CI)	*P *value
					
	Negative (n = 3196)	False positive (n = 2825 )				
**Demographic**						
**Sex**						
Female	1696 (53.07)	1500 (51.89)	1.00	0.36	1.00	0.27
male	1466 (46.93)	1359 (48.11)	1.05(0.95-1.16)		1. 07 (0.95-1.19)	
**Age**						
≤15	875 (27.38)	420 (14.87)	1.00		1.00	
16-25	186 (5.82)	143 (5.06)	1.60 (1.25-2.05)	<0.001	1.38 (0.97-1.97)	0.08
26-35	230 (7.20)	330 (11.68)	2.99 (2.44-3.67)	<0.001	2.21 (1.47-3.33)	<0.001
36-45	609 (19.06)	828 (29.31)	2.83 (2.42-3.31)	<0.001	1.85 (1.26-2.74)	<0.05
46-55	616 (19.27)	601 (21.27)	2.03 (1.73-2.39)	<0.001	1.22 (0.82-1.82)	0.32
56-65	680 (21.28)	503 (17.81)	1.54 (1.31-1.82)	<0.001	0.93 (0.63-1.38)	0.72
**Occupation**						
farmer	1943 (60.79)	1874 (66.34)	1.00		1.00	
fisherman or boatman	135 (4.22)	290 (10.27)	2.23 (1.80-2.76)	<0.001	1.75 (1.40-2.17)	<0.001
student	888 (27.78)	442 (15.65)	0.52 (0.45-0.59)	<0.001	0.97 (0.67-1.40)	0.87
others	230 (7.20)	219 (7.75)	0.99 (0.81-1.20)	0.90	0.98 (0.79-1.20)	0.82
**Education**						
illiteracy	556 (17.40)	506 (17.91)	1.00		1.00	
< high school	2454 (76.78)	2125 (75.22)	0.95 (0.83-1.09)	0.47	0.98 (0.84-1.15)	0.81
high school	76 (2.38)	81 (2.87)	1.17 (0.84-1.64)	0.36	1.05 (0.73-1.53)	0.79
college and above	110 (3.44)	113 (4.00)	1.13 (0.85-1.51)	0.41	1.36 (0.99-1.86)	0.06
						
**Disease factors**						
**History of schistosome infection**						
No	1614 (50.50)	783 (27.72)	1.00	<0.001	1.00	<0.001
Yes	1582 (49.50)	2042 (72.28)	2.70 (2.42-3.01)		2.47 (2.19-2.79)	
**Other helminthes infection**						
no	3053(95.53)	2711 (95.96)	1.00	0.40	1.00	0.50
yes	143(4.47)	114 (4.04)	0.90 (0.70-1.15)		0.91 (0.70-1.19)	

OR : Odds ratio, 95% CI: 95% confidence intervals

In the adjusted multivariate analysis, individuals aged 26-35 (adjusted OR: 2.21, 95% CI: 1.47-3.33) and 36-45 years old (adjusted OR: 1.85, 95% CI: 1.26-2.74) were at significantly greater risk for a false positive result (*P *< 0.05), compared to the group of 6-15 years old. And fisherman or boatman was a risk factor to cause the false positive results of DDIA with adjusted OR of 1.75 (95% CI: 1.40-2.17). The false positive rate of DDIA was significantly higher if the individual was infected with schistosomes in the past (OR = 2.47, 95% CI: 2.19-2.79) (Table 3). Sex, education, and infection with soil-transmitted helminthes were not associated with the false positivity of DDIA in either the bivariate or multivariate analysis.

## Discussion

Although vaccination is thought to be an effective supplemental method for control of schistosomiasis, there is no effective vaccine available to humans at this point and chemotherapy is still the main strategy for reducing the number of schistosomiasis cases [27-30]. Following the recent significant decreases of prevalence and intensity of schistosome infection in P.R. China, the treatment strategy has been transferred from mass chemotherapy to selective chemotherapy for schistosomiasis control [2,31,32]. The infection intensity of patients in our study was very low, and the prevalence of schistosome infection was in the range of 0.38% to 8.23%. The results proved further that the endemic areas of schistosomiasis japonica in P.R. China are characterized by low prevalence and intensity of schistosome infection, after long-term implementation of national control programs [13,14].

To identify individuals to be targeted for chemotherapy in endemic areas, test-treat is probably the most cost-effective approach [33]. Kato-Katz technique is less sensitive for detection of intestinal schistosomiasis in areas of low endemicity, especially in individuals with low infection intensity [5,34]. Immunological assays, with great advantages such as simplicity, rapidity and high sensitivity, have been used to complement stool examinations in P.R. China [13,33]. DDIA is one of few commercially available immunoassays for schistosome infection diagnosis on the market in P.R. China and has been applied in the field for schistosomiasis control based on its quick response and convenience as compared with stool examinations. The practical performance and utility of DDIA in low endemic areas remains unclear. In this study, we evaluated the performance of DDIA, as a primary approach for monitoring the human population in real settings with low endemicity. To evaluate the validity of DDIA, we took the stool examination with the Kato-Katz method and complemented it with the miracidium hatching technique to provide higher sensitivity in parasitological diagnosis of *S. japonicum *infection in our study [9,10].

Previous studies conducted in laboratory using well-archived serum samples and in high endemic areas reported the sensitivities of DDIA ranging from 94.1-100% for detection of acute or chronic schistosomiasis [21,22]. Our study showed that DDIA performed with high sensitivity (91.29%) for the whole population in the present areas, although the value reported here is lightly lower than those in previous studies. No significant difference was detected among villages, by age and gender, or even among the subgroups of infection intensity. These data indicated that DDIA is a stable and highly sensitive diagnostic assay. It should be mentioned that 91.30% (21/23) of misdiagnosed cases by DDIA had infection intensities lower than 100 EPG. Thus more sensitive diagnostic assays or methods should be explored to be used in the endemic areas with very low infection intensity where the missed cases will be of great importance for the transmission of schistosomiasis.

The specificity of DDIA was moderate for the whole population (53.08%), which is much lower than former reports on the same test [21,22,35]. We also found the specificities of DDIA varied in communities and across age strata. This could be explained by multiple factors like population background and immune status, the clinical (disease) status and the socio-demographic characteristics of the study participants. Normally the low specificity of an immunological assay can be explained by (1) cross-reactivity with other helminthes, (2) collection of antibodies of past and current exposure, and (3) insensitivity of stool examination [18,36,37]. In this study, we proved that the low specificity of DDIA was not caused by cross-reactivity with other helminthes, since only 4.44% individuals were infected with soil-transmitted helminthes, and no individual was infected with *Paragonimus *or *Clonorchis *which have been reported to have high cross-reactivity with schistosomiasis immunodiagnostics [19,23]. Multivariate logistic regression analysis in the present study also supported that soil-transmitted helminthes do not influence the specificity of DDIA. The low specificity of DDIA could arguably be due to the well-known insensitivity of single stool Kato-Katz thick smears since the communities in this study were characterized with low prevalence and low infection intensity. Increased stool sampling by obtaining more consecutive stools or combined other different diagnostic methods could better define "true" infected cases and increase the specificity of DDIA [5,37]. In our study, multivariate logistic regression analysis showed that the factors significantly associated with the false positive results of DDIA were occupation, age and history of infection. These data support the idea that the antibody-based immunological test provided the indirect proof of past exposure and reflected the history of schistosome infection, which limits its clinical value for confirmation of cases and the success of treatment [33,37].

Predictive values are the main index to assess the yield value of the screening test when it is used in the field [38]. In our study, DDIA had excellent NPV and varied slightly in the range of 98.64 (95% CI: 97.32-99.96%) - 99.85 (95% CI: 99.57-100.00%), although it was significantly associated with the prevalence of the communities. Those data indicated the high probability of truly uninfected individuals among DDIA negatives in the present population. In areas with low prevalence of infection where selective chemotherapy strategy is carried out, if individual tested by DDIA was negative, no further evaluation or treatment needs to be performed. PPV of DDIA was fairly low with a mean value of 7.86% (95% CI: 6.91-8.81%) and decreased with the prevalence rate determined by stool examination as expected in the low transmission areas. Thus to avoid over-chemotherapy of individuals who have been cured after treatment but still present as antibody positive, a confirmatory method is needed to verify the positive results determined by DDIA [19]. Multiple stool examinations, a combination of different methods, or higher sensitivity and absolute specific molecular methods should be considered to confirm the DDIA positives[5,37-40].

Apart from high sensitivity and specificity, an ideal diagnostic test for use in developing countries should have certain operational characteristics such as being easy to use, rapid and robust, equipment-free and deliverable to end-users [41]. Unlike the CCA-urine dipstick for diagnosing *S. mansoni *infection, the type of detected specimen of DDIA is serum, which needs to be separated from blood cells through centrifuging or by natural separation by putting blood in low temperature for several hours [20,42]. But DDIA meets these criteria to a large extent. The specimen volume needed for DDIA analysis is 20 μl, which can be obtained through finger or earlobe prick. The test procedure is less than five steps and could be operated without extra equipment except a micropipette. The results can be obtained in 5-10 minutes and read by the naked eye. DDIA is portable and can be stored at room temperature for a long time since former studies reported that the performance of DDIA, including sensitivity, specificity, precision were unchanged at 180 days after being stored at 37°C and 360 days after being stored at room temperature [43]. All of these characteristics proved that DDIA could be used in on-the-spot survey in areas of low endemicity. Although the price of DDIA is less than $1 per test, further study needs to be done for analyzing the cost-effectiveness of DDIA when used as a screening test in low endemic areas. This will be great of value to decision makers in budget-planning for schistosomiasis control program.

## Conclusions

Our findings revealed that DDIA presented high sensitivity and NPV in low endemicity areas. We also observed that the moderate specificity of DDIA was mainly caused by the insensitivity of stool examination and high exposure history to schistosomes. As a rapid, simple, apparatus-free and sensitive assay, DDIA is of practical and feasible use to be applied as a screening tool in areas of low endemicity for *S. japonicum*.

## Competing interests

The authors declare that they have no competing interests.

## Authors' contributions

JX, RWP and XNZ designed the study and participated in data collection. JX and XNZ analyzed the data and wrote the first manuscript. DDL, QZW, LT, TF, XHW and JGG organized the field survey and sample examination. All authors contributed to the manuscript and approved its final version.
